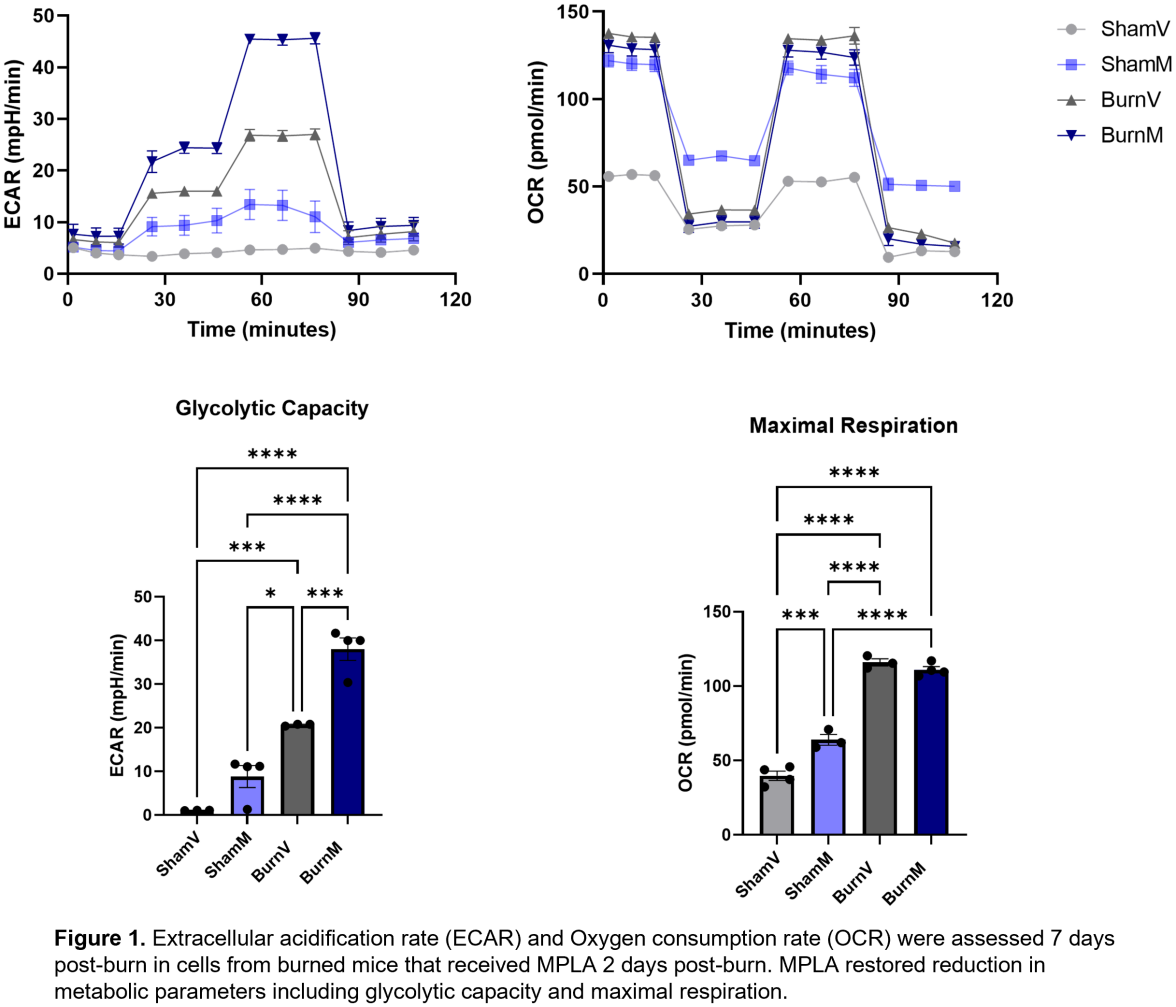# 66 TLR-Mediated Trained Immunity Augments Leukocyte Metabolic Function After Burn

**DOI:** 10.1093/jbcr/iraf019.066

**Published:** 2025-04-01

**Authors:** Mary Oliver, Xenia Davis, Julia Bohannon, Edward Sherwood

**Affiliations:** Vanderbilt University; Vanderbilt University Medical Center; Vanderbilt University Medical Center; Vanderbilt University Medical Center

## Abstract

**Introduction:**

Nosocomial infections affect over 100 million people globally, costing the US $9.8 billion annually in inpatient care alone. Severe burn injuries significantly increase infection risk by impairing both innate and adaptative immune cell function. Our prior research has shown that trained immunity may offer protection against infections after burn. Trained immunity, induced by toll-like receptor (TLR) agonists, promotes sustained metabolic, antimicrobial, and epigenetic alterations in innate leukocytes, priming them for robust, non-specific responses that provide broad protection against diverse pathogens. We hypothesize that treatment with TLR agonists can reverse burn-induced immunosuppression by enhancing metabolic and antimicrobial reprogramming in monocytes and macrophages. This project aims to assess how severe burn affects innate immune cell function in mice, with and without TLR agonist treatment, and to refine these assays in human leukocytes for future burn patient studies.

**Methods:**

In our mouse model, BALB/C mice with a 30% total body surface area (TBSA) scald burn were administered 20 mg of TLR4 agonist monophosphoryl lipid A (MPLA) intraperitoneally 2 days post-burn. On day 7, a subset of mice received intraperitoneal infection with 1x108 CFUs of Pseudomonas aeruginosa. Peritoneal cells were harvested 6 hours later for flow cytometry, Seahorse assays, and Single Cell Energetic metabolism by profiling Translation Inhibition (SCENITH) assay. Plasma cytokines were measured by MagPix multiplex analysis. Additionally, human monocytes from healthy volunteers and a single burn patient (with >20% TBSA burn) were trained with 40 μg/ml MPLA for 24 hours. Seahorse and SCENITH analyses were performed immediately after MPLA exposure and 3 days after its removal to assess metabolic changes.

**Results:**

Burn-injured mice displayed impaired glucose and mitochondrial dependence in monocytes and macrophages, compared to sham controls. However, these impairments were reversed by MPLA training. Flow cytometry revealed reduced expression of immunosuppressive marker CD38 in MPLA-treated cells, while vehicle-treated cells maintained elevated levels. Burn and infected mice also showed elevated inflammatory cytokines, which were reduced by MPLA. Human monocytes treated with MPLA showed increased glycolytic and oxidative capacities, consistent with SCENITH results indicating higher glucose and mitochondrial dependence.

**Conclusions:**

In conclusion, burn injury impaired glucose and mitochondrial dependence in macrophages and monocytes, and increased immunosuppressive markers, all of which were reversed by MPLA training. MPLA-trained human monocytes also demonstrated effective metabolic reprogramming.

**Applicability of Research to Practice:**

TLR agonists represent a promising immunomodulatory strategy for safeguarding burn patients from life-threatening infections.

**Funding for the Study:**

N/A